# A Novel Adaptive Cluster Based Routing Protocol for Energy-Harvesting Wireless Sensor Networks

**DOI:** 10.3390/s22041564

**Published:** 2022-02-17

**Authors:** Bing Han, Feng Ran, Jiao Li, Limin Yan, Huaming Shen, Ang Li

**Affiliations:** 1 Department of Mechatronics Engineering and Automation, Shanghai University, Shanghai 200444, China; hanbing_SHDX@shu.edu.cn (B.H.); ranfeng@shu.edu.cn (F.R.); yanlm@shu.edu.cn (L.Y.); 2 Shanghai Institute of Technical Physics Academy of Sciences, Shanghai 200083, China; 3 Key Laboratory of Infrared System Detection and Imaging Technology, Chinese Academy of Sciences, Shanghai 200083, China; 4 Shanghai Spaceflight Electronic Communication Equipment Research Institute, Shanghai 201109, China; huamingshen@yahoo.com; 5 The College of Information, Mechanical and Electrical Engineering, Shanghai Normal University, Shanghai 201418, China

**Keywords:** wireless sensor network, energy harvesting, hierarchical clustering algorithm, environment adaptive routing protocol, uninterrupted target coverage

## Abstract

With the various applications of the Internet of Things, research into wireless sensor networks (WSNs) has become increasingly important. However, because of their limited energy, the communication abilities of the wireless nodes distributed in the WSN are limited. The main task of WSNs is to collect more data from targets in an energy-efficient way, because the battery replacement of large amounts of nodes is a labor-consuming work. Although the life of WSNs can be prolonged through energy-harvesting (EH) technology, it is necessary to design an energy-efficient routing protocol for the energy harvesting-based wireless sensor networks (EH-WSNs) as the nodes would be unavailable in the energy harvesting phase. A certain number of unavailable nodes would cause a coverage hole, thereby affecting the WSN’s monitoring function of the target environment. In this paper, an adaptive hierarchical-clustering-based routing protocol for EH-WSNs (HCEH-UC) is proposed to achieve uninterrupted coverage of the target region through the distributed adjustment of the data transmission. Firstly, a hierarchical-clustering-based routing protocol is proposed to balance the energy consumption of nodes. Then, a distributed alternation of working modes is proposed to adaptively control the number of nodes in the energy-harvesting mode, which could lead to uninterrupted target coverage. The simulation experimental results verify that the proposed HCEH-UC protocol can prolong the maximal lifetime coverage of WSNs compared with the conventional routing protocol and achieve uninterrupted target coverage using energy-harvesting technology.

## 1. Introduction

Wireless sensor networks (WSNs) are composed of several sensor nodes, which can collect data from the deployment environment and transmit to the gateway through energy-efficient communication for further monitoring or processing. WSNs have a wide range of applications in the field of the Internet of Things, such as smart home, monitoring, and industrial diagnostics. Target coverage is one of the most important performance indicators for WSNs [1], which reflects the network coverage capabilities of wireless sensor nodes, which would directly affect data collection. When the available sensing coverage of WSNs is below the threshold coverage [2], the network would be considered dead (due to the monitoring function failure). Simultaneously, the node topology [3], the communication protocol, and the traffic load [4,5], will affect the energy consumption of nodes. Information reception and transmission is the main cause [6]. Therefore, the balance of traffic load is the crucial task to preserve the hotspot nodes from being quickly exhausted [7,8], thus prolonging the maximum lifetime coverage (MLC) of WSNs. The design of communication protocols can significantly optimize the MLC problem of WSNs.

Many research works exist on data transmission schemes used to balance the energy consumption of WSNs and the data compression methods used to reduce the energy required for data transmission [9,10]. However, data fusion is a comprehensive problem, which contains many problems that remain to be studied. Therefore, the optimization of routing protocols is the main focus of this paper, and will be comprehensively reviewed in this section. The data sensed by the nodes are transmitted to the base station (BS) [11] to complete the monitoring of the target area. For the transmission of data of the same size, the energy consumption obviously increases along the propagation distance, especially for the scenario where the base station is fixed far from the information collection network. Thus, the heavy transmission task may cause a degradation in the perceived quality of the sink nodes and queuing delays due to insufficient bandwidth [12]. A comparison of the conventional routing protocols is depicted in Table 1.

In [13], a low-energy adaptive clustering hierarchy LEACH) was proposed, the cluster head nodes (CHs) compress the data received from the respective cluster and send an aggregated packet to the BS to reduce the amount of transmission data [14]. In [15], a greedy, chain-based, power-efficient gathering in sensor information systems (PEGASIS) is proposed to optimize the LEACH protocol. The PEGASIS protocol organizes all of the nodes to form a chain, so the data can be fused hierarchically and transmitted to the BS by the leader head node. To optimize the energy-efficient routing protocol based on these widely-used classic protocols, a centralized energy efficient distance (CEED) routing protocol [16] is proposed to establish the chain among the specifically formed CHs to evenly distribute the energy consumption of all sensor nodes. In [17], a BS-centralized energy regulation has been added to the LEACH protocol to form the LEACH-centralized (LEACH-C) protocol, which can avoid nodes with low energy being selected as the cluster head. A parallel communication structure is designed for the PEGASIS protocol in [18] to reduce the transmission delay caused by the chain-based routing protocol. However, the stored energy of the node would inevitably be exhausted and cause a coverage hole. Thus, the EH technology [19] is an excellent option to guarantee uninterrupted coverage [20] of the network.

In the EH-WSNs, each sensor node possesses the ability to capture energy from the environment into its own rechargeable battery, such as wind, solar, and thermal energy [21]. For the whole-energy management of WSNs, uninterrupted coverage can be accomplished when the amount of energy harvesting is more than or equal to the energy consumption [22]. Therefore, the MLC of WNSs not only depends on EH efficiency, but also the energy consumption [23]. The WSN node can not perform data transmission in the energy-harvesting phase, so the energy-efficient protocol should be redefined for the EH network to deal with the variable information routing and the inoperative nodes [24]. In [25], a novel energy-harvesting clustering protocol (NEHCP) is proposed based on the hierarchical clustering routing protocol. The collected data are aggregated and transmitted to the base station under uncontrollable ambient resources based on EH technology. The energy-efficient protocol was improved by the Euclidean distance matrix reconstruction method in [26], which could solve the intermittent energy shortage caused by the imbalance between harvested and demanded energy in EH-WSNs. Although the energy can be harvested, these research achievements prove that energy-consumption optimization remains an important problem in EH-WSNs. As mentioned above, the cluster-based and the chain-based routing protocol possess the ability to accomplish the energy-efficient WSNs through the optimization of the data transmission. However, the node in EH-WSNs would be inoperative in the EH phase, as the disabled node may completely paralyze the chain-based routing if the distance between the last node and the next node is beyond the communication distance. Thus, for the routing protocol design for EH-WSNs, the cluster-based routing protocol has become the research focus because of the independent node assignment and the robust cluster reconstruction.

In the cluster-based routing protocol of EH-WSNs, the node-clustering method and the management of CHs remain the essential problems. In [27], a triangular, fuzzy-based, spectral cluster routing (TF-SCR) mechanism is proposed, considering the MLC of WSNs and the reliability of data transmission. The spectral clustering method is adopted to accomplish the residual-energy-based node clustering. Simultaneously, the triangular fuzzy membership function is applied to choose the node with the higher signal strength and more residual energy as the cluster head. The data packets are aggregated to the chosen cluster head and transmitted to the sink node with the minimum routing overhead. In [28], a particle swarm optimization (PSO) algorithm is adopted in the design of the routing protocol to minimize the intra-cluster distance and thus accomplish the energy-efficient routing protocol. In [29], a fuzzy-enhanced flower pollination algorithm-based, threshold-sensitive, energy-efficient clustering protocol is proposed to optimize the cluster head selection method using the heuristic algorithm. The sensor parameters, including the residual energy, the node centrality, and the distance to BS, are collaboratively considered in the determination of the cluster head node. The existing stable election protocol (SEP) in [30] is optimized to maintain a uniform energy distribution between cluster head nodes and member nodes. Different residual energy thresholds are proposed for the node with different energy states, which can determine the criteria needed to reform the cluster and reselect the CHs. The extra energy consumption in the unnecessary reconstruction of clusters can be restricted. The clustering algorithm and the selection of the cluster head are the crucial aspects of the clustering-based routing protocol. Different clustering methods would directly influence the energy consumption of intra-cluster data transmission. In recent years, the centralized energy-efficient cluster (CEEC) [31], the hybrid unequal clustering layering protocol (HUCL) [32], and the sleep–awake energy-efficient distributed (SEED) [33] are proposed to guarantee a longer MLC. The optimized selection of the cluster head could improve the energy efficiency of data transmission to the base station and the alternative takeover of the cluster head would balance the energy consumption of nodes to prolong the MLC of the network.

The routing protocols optimized above have considered the transmission load and the energy consumption; several excellent research achievements have been proposed. However, IoTs (Internet of Things) epitomizes the interconnection of things. Therefore, different from the internet, the information from any terminal is indispensable for IoTs. The death of nodes from one region may lead to the loss of complete control of one type of equipment. Therefore, adaptive networking and distributed data routing would be more suitable for the operation of IoTs. In this paper, an adaptive hierarchical-clustering-based routing protocol is proposed for the WSNs with energy harvesting (EH). Aimed towards the specific problem of EH-WSNs, where parts of nodes are in an inoperative status because of energy harvesting, the proposed HCEH-UC fuses the adaptive node-clustering algorithm and the distributed alternative CHs scheme. The environment adaptive node clustering algorithm can form reasonable node clusters according to the original distribution of nodes, thus reducing the influence of human experience on the number or topology of clustering. According to the remaining energy and the topology of the nodes, the data transmission mode is adaptively regulated in a distributed way through the switch of the energy-harvesting mode and the operation mode.

In order to verify the performance of the proposed HCEH-UC protocol, a regular WSNs was constructed and compared with the conventional routing protocols. Simulation results show that the proposed ECEH-UC protocol can accomplish the uninterrupted coverage of target area based on the EH-WSNs through an energy-efficient way.

The main contributions of this paper are summarized as follows:

(1) A novel environment-adaptive clustering algorithm for WSNs nodes has been proposed. The clustering termination condition can be adaptively adjusted according to the node deployment and form a suitable node topology. Therefore, reasonable data transmission and data fusion mode of the WSNs nodes would guarantee energy efficiency.
(2) A data transmission adjustment mechanism is proposed for the EH-WSNs and forms the proposed routing protocol (HCEH-UC). The unique modes of EH-WSNs nodes, including energy harvesting (sleeping-mode) and data transmission (operation-mode), require a suitable succession method. Thus, a corresponding routing mechanism is proposed for EH-WSNs to sustain the uninterrupted coverage of the target area.

The remainder of this paper is organized as follows. Section 2 introduces the proposed HCEH-UC routing protocol, including the environment-adaptive hierarchical clustering algorithm and the distributed data transmission mode adjustment method. Verification simulations are conducted in an emulation WSNs using MATLAB, and an analysis of the comparison results is given in Section 3.

## 2. Adaptive Hierarchical-Clustering-Based Routing Protocol for EH-WSNs

### 2.1. Environment-Adaptive Hierarchical Clustering

The hierarchical clustering (HC) algorithm is a fast-clustering algorithm proposed by Dror et al. [34], which can independently accomplish agglomerative clustering according to node deployment. The clustering phase does not need the cluster head or the number of clusters to be appointed by human beings. In order to optimize the data transmission mode inside each cluster, the agglomerative HC algorithm is adopted and accomplishes a reasonable clustering after the deployment of the WSN nodes. Therefore, in the monitoring phase of WSNs, the data are received from the nodes to CHs and merged into the data packages. These data packages are sent to the base station by CHs, which indicates that the energy consumption of the CHs is greater than the other nodes inside the cluster. Utilization of the clustering algorithm can accomplish the optimization of data routing and guarantee high energy efficiency.

However, the clustering process of the HC algorithm needs to be terminated according to human experience, otherwise all nodes merge into one cluster. The clustering results would determine the data routing of the distributed node cluster, thus influencing the energy consumption of WSNs. To reduce the participation of the human experience, an environment-adaptive hierarchical clustering algorithm is proposed in this paper. The clustering process is terminated according to the adaptive deployment of the nodes and accomplishes the spontaneous clustering of the WSN nodes.

Assume that *M* nodes are optimally deployed in the positioning area, and the coordinates of nodes are fixed and known a priori. According to the theory of the aggregation hierarchical clustering algorithm, these *M* nodes would be regarded as the initial clusters:

(1) $$C_{i}=\left\{X_{i}\right\},i\in M$$

where $C_{i}$ represents the *i*th formed cluster. The largest Euclidean distance between any two clusters would be calculated and adopted as the clustering cost for each clustering iteration. Then, the two clusters with the closest distance would merge into a new cluster until the required number of clusters or the termination condition is reached.

Assuming that, after several clustering process, the cluster $C_{M+a}$ contains $\left\{X_{i},X_{j}\right\}$, and the cluster $C_{M+b}$ contains $\left\{X_{k},X_{l}\right\}$. Among all of the contained nodes, the $X_{i}$ and $X_{k}$ are the nodes that are farther apart. According to the definition of the largest distance between clusters in the HC algorithm, the largest distance $D\left(C_{M+a},C_{M+b}\right)$ between these two clusters can be depicted as (3).

(2) $$D\left(C_{M+a},C_{M+b}\right)=D\left(X_{i},X_{k}\right)$$

where M+a and M+b represent the label of the clusters.

In this paper, the largest distance between the deployed nodes $D_{max}$ is selected and adjusted to serve as the clustering termination threshold *T*.

(3) $$\begin{array}{c} T=\sigma D_{max}\\ =\sigma \max\left\{\sqrt{{\left(X_{i}-X_{j}\right)}^{2}+{\left(Y_{i}-Y_{j}\right)}^{2}}\right\},i,j\in M,i\neq j \end{array}$$

where $X_{i}=\left(X_{i},Y_{i}\right),X_{j}=\left(X_{j},Y_{j}\right)$ denotes the coordinates of the *i*th and *j*th nodes, and σ represents the practical factor, which is defined as the ratio of distance between nodes within the confidence distance.

A detailed description of σ is given here. Firstly, the confidence distance for reliable data transmission can be obtained in advance as $D_{c}$ through the data transmission simulations in the practical target area. Then, the distance between the *i*th node and the *j*th node is calculated and recorded as $d_{ij}$, and the proportion of $d_{ij}<D_{c}$ could be calculated and recorded as σ. Thus, the deduced proportion σ could participate in the calculation of the threshold *T* according to (3).

The clustering termination is synergistically decided by the maximum distance and reliable data-transmission distance between the deployed nodes, which can guarantee a difference between clusters and reduce the difference inside the formed cluster. Therefore, the fusion of the confidence distance and the node topology could accomplish a more reasonable node clustering.

To transfer a certain amount of data to the base station, the energy consumption of each cluster would increase with the increase in the threshold distance. However, the number of clusters would decrease accordingly, and reduce the transmission amount of the data package. The optimization of the threshold distance could be meaningful when improving the energy efficiency. Therefore, the proposed environment-adaptive hierarchical clustering could increase the MLC of WSNs by optimizing the node topology.

### 2.2. HCEH-UC Routing Algorithm

The clustering-based routing algorithm, such as the low-energy adaptive clustering hierarchy (LEACH) [11], can dynamically establish clusters and randomly select CHs in every cycle to balance the energy consumption of the nodes and prolong the MLC of WSNs. However, nodes’ energy would finally be exhausted without energy harvesting. For the EH-WSNs, the node can hardly operate in the energy-harvesting mode. Thus, the design of the transmission modes based on the environment-adaptive node cluster is also an important problem when accomplishing uninterrupted network coverage.

Aiming to optimize the data transmission mode of the WSN nodes, a distributed data transmission mode adjustment method is proposed in this paper. The cluster head node could be alternated according to the remaining energy and the exhausted node would be re-charged in time to prepare for the next cycle. Each cluster could adaptively accomplish the distributed control of the data transmission mode to limit the number of sleeping nodes in each data collection cycle, which can guarantee the normal operation of WSNs with high-energy efficiency. Thus, the uninterrupted coverage of the target area for EH-WSNs can be accomplished by the proposed HCEH-UC routing algorithm.

To calculate the energy consumption of data transmission in the WSNs, the radio energy consumption model proposed in [15] is adopted in this paper, as shown in Table 2.

The amplification constants, $\epsilon_{\mathrm{fs}}$ and $\epsilon_{\mathrm{mp}}$, represent energy consumption when amplifying the signal, which can support data transmission to a certain distance. Therefore, these constants are related to the size of the transmission data and the transmission distance. The energy consumption of the radio transmission and reception mode can be denoted as $E_{elec}=50\cdot \mathrm{nJ}/\mathrm{bit}$. According to the channel transmission model, the energy consumption of the transmission would be the square of the distance.

According to the literature [17], the free space and multipath attenuation models are used to establish a wireless channel propagation model, and the energy consumption $E_{Tx}$ for sending k−bit data can be described as (4):

(4) $$\begin{array}{cc} E_{Tx}\left(k,d\right) & =E_{Tx-elec}\left(k\right)+E_{Tx-amp}\left(k,d\right)\\ \; & =\left\{\begin{array}{c} E_{elec}*k+\epsilon_{\mathrm{fs}}*k*d^{2},d<d_{0}\\ E_{elec}*k+\epsilon_{\mathrm{mp}}*k*d^{4},d\geq d_{0} \end{array}\right. \end{array}$$

where *d* denotes the transmission distance, $d_{0}$ denotes the distance threshold, $E_{Tx-elec}$ represents the transmission energy, and $E_{Tx-amp}$ represents the amplification energy required for data transmission to the distance *d*:

$$d_{0}=\sqrt{\frac{\epsilon_{\mathrm{fs}}}{\epsilon_{\mathrm{mp}}}}.$$

At the same time, the energy required to receive k−bit data can be depicted as (5):

(5) $$E_{Rx}\left(k\right)=E_{Rx-elec}\left(k\right)=E_{elec}*k.$$

The energy consumption of the cluster head node includes the data transmission and reception, as well as the generation and maintenance of the routing framework, which indicates that the energy consumption of the cluster head node is much higher compared with the other node in the cluster. In order to achieve uninterrupted WSN coverage, the cluster head node needs to sleep and implement energy harvesting after a complete data-transmission cycle to participate in the next cycle. The selection of the successor cluster head node is based on the remaining node energy in the cluster and node location information.

**Figure 1:**
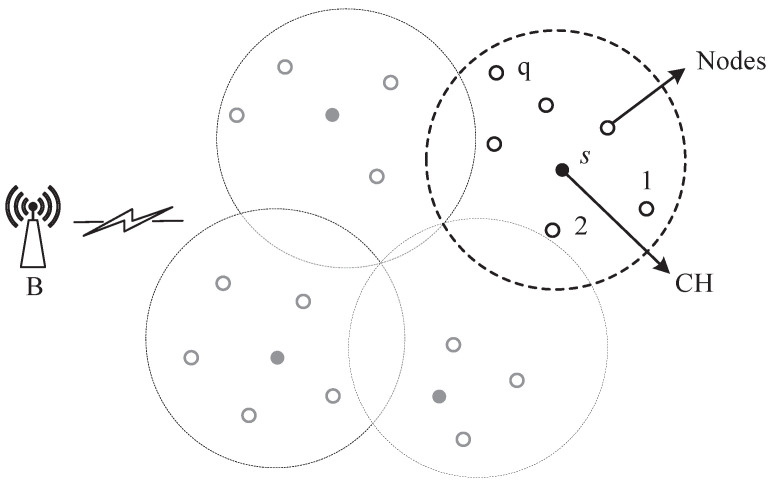
Topology of wireless sensor network.

As shown in Figure 1, the improved hierarchical clustering algorithm was adopted on the WSN nodes for reasonable clustering, and the nodes in the cluster conform to the star topology, which could gather information from the marginal nodes to CHs. Then, CHs compress the received data and finally transmit them to the base station or end user termination. Assuming that a cluster includes *Q* nodes, the base station is represented by *B*, the total amount of transmission data to the base station in one cycle for this cluster is $k_{Bs}$ bit, the distance between the cluster head node and the base station is $d_{Bs}$, the *q*th node in the cluster need to transfer to the cluster head node *s* in each cycle is $k_{qs}$ bit, and the distance between *q* and *s* is $d_{qs}$.

In the network shown in Figure 1, the energy consumption $E_{Bs}$ of the cluster head node *s* in one cycle includes the energy consumption $E_{Rx}$ of receiving the data from the nodes, the energy consumption $E_{Df}$ of data fusion, and the energy consumption $E_{Tx}$ when sending the data package to the base station *B*, which can be described as (6):

(6) $$\begin{array}{cc} E_{Bs} & =E_{Rx}\left(k_{Bs}\right)+E_{Df}\left(k_{Bs}\right)+E_{Tx}\left(k_{Bs},d_{Bs}\right)\\ \; & =E_{\mathrm{elec}}*k_{B_{s}}+E_{DA}*k_{B_{s}}+E_{Tx-elec}\left(k_{B_{s}}\right)+E_{Tx-amp}\left(k_{B_{s}},d\right) \end{array}$$

where $E_{DA}$ represents the energy consumption constant for data fusion.

Assume the node *q* transmits $k_{qs}$ bit data to the cluster head node *s* in one data transmission cycle:

(7) $$\sum_{q=1}^{Q}k_{qs}=k_{Bs}.$$

Thus, the energy consumption $E_{qs}$ of the *q*th node to transfer these data can be described as (8):

(8) $$E_{qs}=E_{Tx-elec}\left(k_{qs}\right)+E_{Tx-amp}\left(k_{qs},d\right),q\neq s.$$

When the battery capacity of the cluster head node is insufficient to support the routine operation, the exhausted cluster head node should alter into the sleep node to collect the energy. The sleep node can barely implement the data delivery mission. Therefore, aiming towards uninterruptible target coverage with energy harvesting (UC-EH), the appropriate node would be selected as the new cluster head node based on the location information and status of the remaining nodes in the cluster. Assume that the $E_{estimation}$ represents the energy consumption of data delivery for different nodes:

(9) $$E_{estimation}\left(s\right)=E_{Bs}+\sum_{q=1}^{Q}E_{qs},q\neq s$$

where the $E_{estimation}\left(s\right)$ includes the energy required $E_{qs}$ for nodes to transmit data to the cluster head *s* and the energy required $E_{Bs}$ for the cluster head node to transmit data to the base station. Assume that the remaining node energy can be represented by $E_{rest}$, and the ρ is adopted to represent the probability of being selected as a cluster head for the *q*th.

(10) $$\rho \left(q\right)=\left\{\begin{array}{c} 1-\frac{E_{estimation}\left(q\right)}{E_{rest}\left(q\right)},q\in G\\ 0\;,q\notin G \end{array}\right.$$

where *G* represents the set of unselected nodes in the current data-transmission cycle. The $E_{estimation}$ would increase along with the increase in the distance from nodes to the cluster head node and the cluster head to the base station. The energy required for data delivery of the successor cluster head should be small and the energy remaining in the successor cluster head should be sufficient, which means that the node with the largest probability ρ becomes the successor CH.

The node clusters formed by the proposed clustering algorithm would be evenly distributed in the target detection area, and the distance between the nodes in the cluster is much smaller than the distance to the base station. Therefore, the energy consumption of the cluster head node is much larger than that of the other nodes. The energy collected by the cluster head needs to support data delivery with the base station and data delivery with the nodes in the cluster, as well as information interaction with the successor cluster head. The proposed HCEH-UC can decrease energy consumption through the optimization of data-routing modes and prolong the MLC of the node network. However, when a certain number of nodes in the network are in a sleep-state, this network would be defined as the network in death and cannot continue to perform the target monitoring function [14].

In this paper, the EH technology is introduced in the proposed HCEH-UC, and an HCEH-UC algorithm is proposed for EH-WSNs to accomplish uninterruptible network node coverage. The energy harvesting mode of each node cluster would be adaptively generated. According to the data-delivery energy model and the cluster head selection mechanism, energy consumption would be optimized while ensuring the routine operation of the network. Assuming that each cluster head node needs to complete at least *Z* times the data transmission tasks within the base station during one cycle, the energy collection of the cluster head node *s* needs to be greater than the threshold $E_{\Delta }$, as shown in (11).

(11) $$E_{\Delta }\geq Z*\left(E_{Bs}+\sum_{q=1}^{Q}E_{qs}\right),q\neq s$$

where $E_{Bs}$ represents the energy consumption when the node *s* is adopted as the CH; $E_{qs}$ represents the energy consumption when transmitting data to the successor cluster head as a non-cluster head node. Assume the required charging time of up to $E_{\Delta }$ is $\sigma_{E}$, and the time required to complete the information collection for one cluster is ΔT. Under certain energy harvesting conditions and the working mode of the cluster head node, the network can run permanently if the network possesses sufficient numbers of sensor nodes. However, the redundancy problem would lead to additional energy consumption in the network. To ensure the routine operation of the network, the required condition can be shown as (12):

(12) $$\sigma_{E}\leq \left(30\%*Q\right)\;*\;\left(Z*\Delta T\right).$$

According to (12), the minimum number *Q* of nodes required to maintain the routine operation of the network according to the node location, communication information, and energy collection efficiency, which could ensure the cluster head node in sleep mode, has enough time for the energy collection to sustain the operation of the WSNs.

External energy supply includes solar energy, wind energy, etc., which will not be 100% converted into electric energy stored by the node. Simultaneously, the data transmission mechanism will also be adaptively adjusted according to the different charging scenarios. If energy supply methods are considered, the corresponding data routing will become a more complicated issue. Therefore, in this paper, the quantity of the electricity collection, instead of the collection efficiency, will be considered.

Therefore, the proposed HCEH-UC routing algorithm can be specifically expressed by the pseudocode, as shown in Algorithm 1.
**Algorithm 1:** HCEH-UC routing algorithm
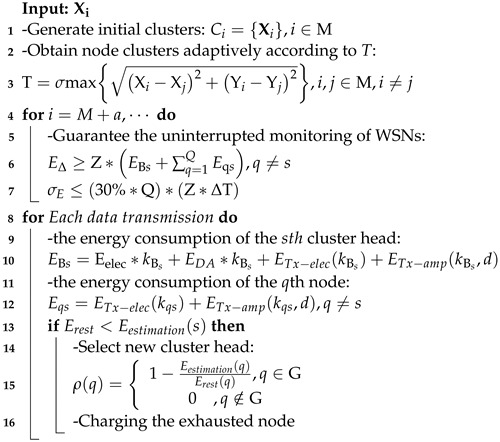


In addition, in the event of unexpected node exhaustion, the proposed HCEH-UC will perform node clustering and form a new node topology according to the existing normal nodes to adaptively obtain a new distributed routing mode. Therefore, the proposed environment-adaptive method can generate a robust information-delivery mode in a distributed way according to the distribution of the network nodes.

### 2.3. Algorithm Complexity Analysis

In the node clustering-based routing algorithm, time complexity mainly exists in the cluster formation phase and the data transmission phase. Therefore, the time complexity of the proposed HCEH-UC routing algorithm also contains two parts, including the adaptive hierarchical clustering of the WSNs nodes and the data transmission control in the formed node cluster. The time complexity of the clustering is $O(M^{2}+M_{clu}+M_{ter})$, where $O(M^{2})$ represents the time complexity of calculating the Euclidean distance between WSNs nodes, $O(M_{clu})$ denotes the time complexity of two clusters converging into one cluster, and $O(M_{ter})$ represents the time complexity of the clustering termination judgment. Assuming that *P* numbers of node clusters are eventually formed, the pth node cluster can stably execute $Z_{p}$ numbers of a data transmission task, the CH selection cost $O(s_{t})$ time complexity, the control information of the data transmission mode requires O(ctr) time complexity, and the time complexity of the data transmission is O(t). Therefore, the time complexity of $Z_{all}$ can be deduced as $\sum_{p\in [1,P]}\left(\left(Z_{all}/Z_{p}\right)\left(O\left(s_{t}\right)+O\left(ctr\right)+Z_{p}*O\left(t\right)\right)\right)$. In conclusion, the time complexity of the proposed HCEH-UC can be depicted as $O\left(M^{2}+M_{clu}+M_{ter}\right)+\sum_{p\in [1,P]}\left(\left(Z_{all}/Z_{p}\right)\left(O\left(s_{t}\right)+O\left(ctr\right)+Z_{p}*O\left(t\right)\right)\right)$. Compared with the contrast clustering-based algorithm [13,17,25], the proposed algorithm requires more time complexity in judging the termination of the clustering. However, it also reduces the frequency of the global CH election. Therefore, the proposed HCEH-UC can improve the energy efficiency without sacrificing the time complexity.

## 3. Simulation Results

**Figure 2:**
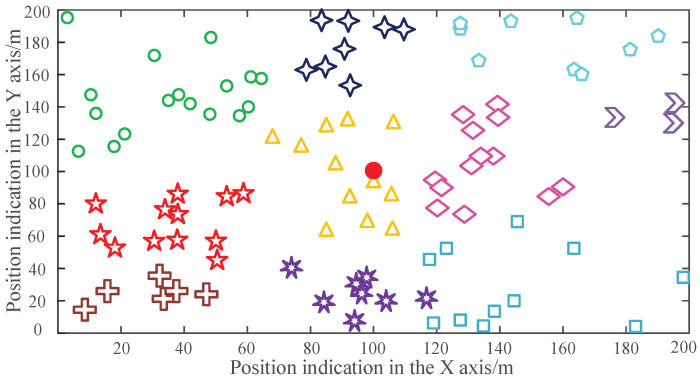
Node distribution of the WSNs.

The HCEH-UC algorithm would be evaluated in the simulated network without/with energy harvesting using MATLAB in this section. MATLAB is also a commonly used verification platform for the contrast routing algorithm. The distribution of 100 sensor nodes in the 200 m × 200 m area is shown in Figure 2. In the practical WSNs, the location of the sensor nodes can be obtained from the deployment of the nodes. Various colors represent different cluster attributions, which will be described in the next section. The initial cluster head could also be appointed or elected. The other simulation parameters are depicted in Table 3. In order to conduct fair comparison experiments, these parameters are consistent with the comparison algorithm [25].

### 3.1. Network Lifetime Evaluation

In this section, the proposed HCEH-UC would be verified through comparison with the conventional routing algorithm including LEACH [11], LEACH-C [17], CEEC [31], HUCL [32], SEED [33], and NEHCP [25]. The conventional routing algorithms do not possess an energy-harvesting phase. To obtain the equal and effective contrast experimental results, the HCEH-UC would first be verified without energy harvesting to prove its ability to improve energy efficiency.

For different routing algorithms, the data transmission mechanisms are different from each other. Even for the clustering-based routing algorithm, the 100 sensor nodes could be clustered through various methods and lead to different data fusion modes. The cluster results of the proposed environment-adaptive hierarchical clustering algorithm are shown in Figure 2. Each shape in the figure represents one node-cluster and the solid circle denotes the base station.

**Figure 3:**
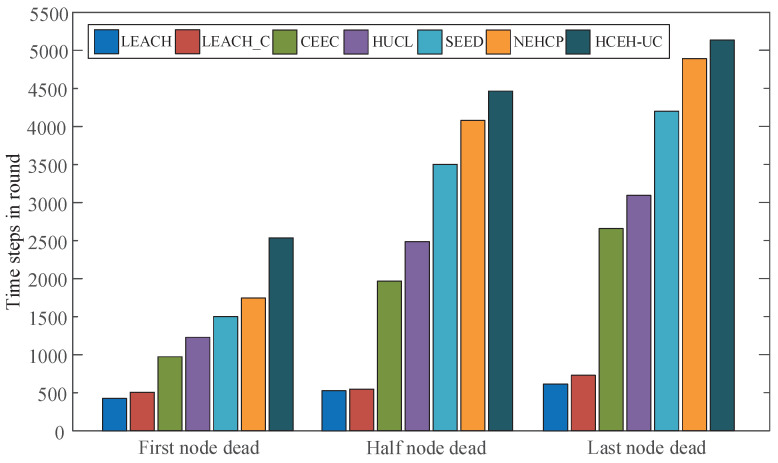
Data transmission rounds before the first node dead (FND).

The results of clustering would directly influence the final results and the data transmission mechanism. Additionally, the cluster formation would also spend some non-negligible energy. Thus, the optimization of the clustering algorithm and the adaptive transformation would be meaningful research. In the verification experiments, the number of data transmission rounds performed when the first node dead (FND), the half node dead (HND), and last node dead (LND) of all routing algorithms are recorded in Figure 3. The corresponding quantitative comparison of the metrics are shown in Table 4. These crucial metrics would be directly compared to evaluate the performance of different routing algorithms.

As shown in Figure 3, the conventional routing algorithm LEACH and LEACH-C can prolong the FND index to 452 and 513, respectively. The CEEC, HUCL, and SEED routing protocol can prolong the FND to 1000, 1250, and 1510, respectively. The advanced NEHCP protocol leverages the clustering algorithm and extends the FND to 1756. The HCEH-UC that is proposed in this paper can accomplish the optimized clustering of the WSNs nodes through the environmental-adaptive clustering algorithm, which can reduce the energy consumption inside the cluster and balance the energy consumption of the network. Therefore, the proposed algorithm can prolong the FND rounds to 2535 rounds.

Simultaneously, as shown in Table 4, the superior lifetime metrics, including HND and LND, when compared with the conventional protocol, can also verify the proposed HCEH-UC, which means that the proposed routing algorithm can accomplish a more energy-efficient node topology through an environment-adaptive clustering algorithm. Therefore, an optimized data-routing mode could reduce the energy consumption needed for data transmission.

### 3.2. Stability Period and Instability Period Evaluation

**Figure 4:**
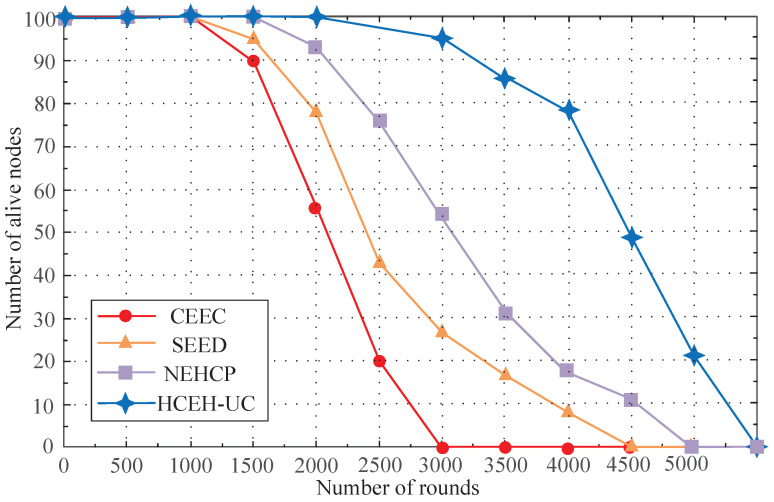
Number of alive sensor nodes per round for different routing protocols.

To conduct a more comprehensive comparison, the metrics of the stability period and instability period need to be evaluated. The protocol with a superior performance, including the CEEC, SEED, and NEHCP protocols, was chosen to obtain clearer figure results. The number of alive sensor nodes for these contrast algorithms was recorded in each round and is depicted in Figure 4.

Assume that the network with no exhausted nodes is defined as the stable phase, and the network with exhausted nodes is defined as the unstable phase. As shown in Figure 4, the proposed HCEH-UC possesses a longer stable phase. The LND of the network was also extended, which means that more data-transmission tasks can be executed.

### 3.3. MLC Evaluation with Various Initial Energy

**Figure 5:**
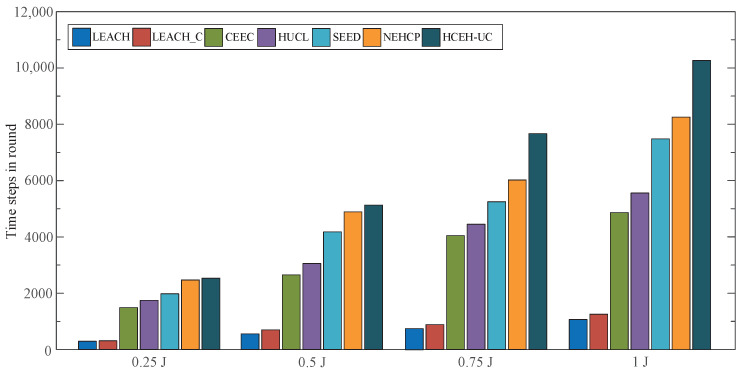
MLC of different routing protocols under various energy levels.

In order to perform the conventional routing protocol in various scenes, the energy levels would be set to 0.25 J, 0.5 J, 0.75 J, and 1 J in different scenes. The MLC of the conventional algorithms are depicted in Figure 5, and a quantitative comparison of the metrics is given in Table 5.

As shown in Figure 5 and Table 5, with the absence of energy harvesting, the MLC of the network would be prolonged, along with the increase in the initial node energy. Simultaneously, the proposed HCEH-UC has a superior performance at various energy levels compared with the contrast routing protocols.

### 3.4. Relationship between Energy Consumption and Energy Harvesting

**Figure 6:**
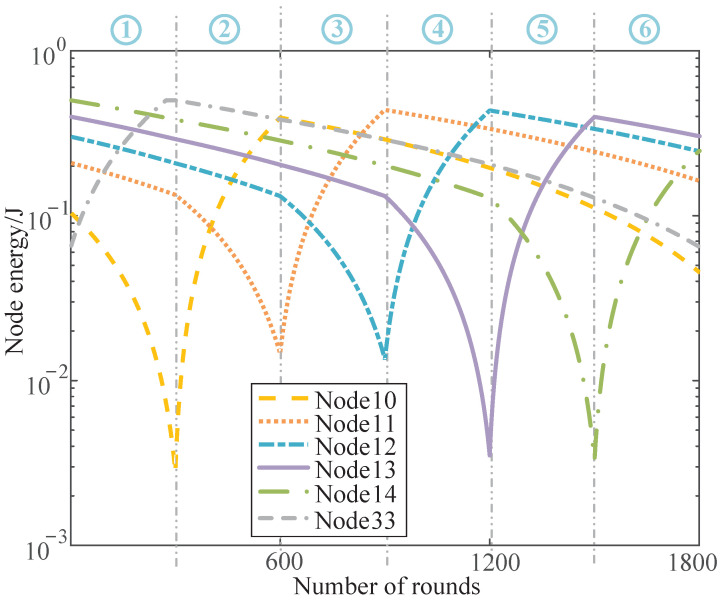
Residual energy of the node with energy harvesting.

The network with limited energy capacity can be optimized using the proposed HCEH-UC. However, the monitoring function of the target area is finally invalid when a certain number of the nodes are exhausted. With the addition of the energy-harvesting technique, the proposed HCEH-UC can accomplish the uninterrupted coverage of the target area. The proposed HCEH-UC algorithm can adaptively adjust the distributed communication mode of clusters according to the topology relationship and the remaining energy of the cluster nodes. Thus, the working and sleep modes of the cluster head could be managed and the number of nodes in sleep mode can be controlled to accomplish energy harvesting. In order to demonstrate the effectiveness of the proposed HCEH-UC algorithm under the energy-harvesting condition, the remaining energy changes with the number of data-transmission rounds for one arbitrary cluster is shown in Figure 6, and the ordinate axis is set to a logarithmic axis in order to intuitively indicate the energy variation tendencies.

Additionally, the energy consumption and collection situation of the nodes is depicted in Figure 6. Each energy collection and consumption cycle was divided into three stages, including the cluster head phase, data transmission to cluster head phase, and sleep and energy harvesting phase. When the residual energy is sufficient, the node alternatively acts as the cluster head or the data-transmission node, and the exhausted node would switch into sleep mode and implement energy harvesting when the energy is insufficient. Therefore, uninterrupted target coverage can then be accomplished based on energy harvesting.

Take a complete cycle from the residual energy curve for specific analysis. The example cluster contains six nodes, including nodes 10, 11, 12, 13, 14, and 33. In the first stage, node 10 of the cluster serves as the cluster head node, nodes 11, 12, 13, and 14 serve as data-transmission nodes, and node 33 enters sleep mode due to insufficient energy and performs energy harvesting. At this stage, the energy of node 10 drops rapidly, nodes 11, 12, 13, and 14 drop slightly, and the energy stored in node 33 rises steadily.

In the second stage, node 11 would be selected as the cluster head based on the remaining energy and node information through the cluster-head-selection mechanism proposed in this paper. Cluster head node 10 would switch into sleep mode and collect energy due to the insufficient energy. Nodes 12, 13, 14, and node 33, which has completed energy storage, would serve as the data-transmission nodes until the energy of node 11 reaches a low level. When the energy harvesting of node 10 is completed, node 11 would switch into sleep mode and the successor cluster head node would be re-elected; thus, the third stage and the following stage can start.

The energy consumption of cluster head nodes is relatively high, and the energy consumption of non-CH nodes is relatively slow. When the energy storage is insufficient, the node switches into a sleep node to accomplish energy harvesting. The simulation results prove that the algorithm proposed in this paper can control the number of nodes in the sleep mode for one cluster within a reasonable range through the reasonable adjustment of the communication mode of nodes. Therefore, uninterrupted target coverage would be accomplished based on energy harvesting.

## 4. Conclusions

This paper proposes an energy harvesting-based routing algorithm for uninterrupted WSN target coverage. Firstly, based on the proposed hierarchical clustering algorithm, the nodes can accomplish environment-adaptive clustering based on the distance between each other, which can reduce the energy consumption of data transmission inside the cluster and optimize the topological relationship of the network. A cluster head selection mechanism is then proposed based on three crucial aspects of energy harvesting, including the residual energy of nodes, the data-transmission energy model, and the energy collection needed to form an advanced HCEH-UC routing protocol. Finally, the distributed communication mode and topological relationship of the node cluster that is formed can be adaptively determined through the alternative operating–recharging mode of the cluster head node. Therefore, an energy harvesting-based, uninterrupted target coverage can be accomplished. In order to verify the proposed HCEH-UC routing algorithm, comparison simulations were conducted in terms of the improved clustering algorithm and the energy harvesting-based, uninterrupted target coverage algorithm. Compared with the conventional routing algorithm, the simulation results proved the effectiveness of the proposed HCEH-UC routing algorithm.

## Figures and Tables

**Table 1:** Comparison of the conventional routing protocols.

Protocol	Structure	Feature
Low-energy adaptive clustering hierarchy (LEACH) [13]	Clustering	The cluster head nodes compress data received from the respective cluster and send an aggregated packet to the base station in order to reduce the amount of transmission data
LEACH-centralized (LEACH-C) [17]	Clustering	The base station centralized energy regulation is added to avoid nodes with low energy being selected as the cluster head, thus prolonging the maximum lifetime coverage (MLC) of wireless sensor networks
Power-efficient gathering in sensor information systems (PEGASIS) [15]	Chain	Organizes all of the nodes to form a chain, which is constructed by some specific node according to the nearest-neighbor principle
Centralized energy efficient distance (CEED) [16]	Chain	Establish the chain among the specifically formed cluster heads to evenly distribute the energy consumption of all sensor nodes

**Table 2:** The energy consumption in radio transmission and reception mode.

Mode	Energy-Consumption
transmission/reception mode	$50(\mathrm{nJ}\cdot {\mathrm{bit}}^{-1})$
free-space information amplification ($\epsilon_{\mathrm{fs}}$)	$10(\mathrm{pJ}\cdot {\mathrm{bit}}^{-1}\cdot m^{-2})$
multipath-fading information amplification ($\epsilon_{\mathrm{mp}}$)	$0.0013(\mathrm{pJ}\cdot {\mathrm{bit}}^{-1}\cdot m^{-4})$

**Table 3:** Simulation parameters.

Parameters	Value
Sensor Network Size	200 m × 200 m
Nodes Number	100
Base Station	(100,100)
Initial Energy	0.5 J
Data-Packet Size	4000 bit
Packet Header Size	25 bytes
Control Message Size	50 bytes
$E_{elec}$	50 nJ/bit
$E_{fs}$	10 pJ/bit/m${}^{2}$
$E_{mp}$	0.0013 pJ/bit/m${}^{4}$
$E_{DA}$	5 nJ/bit/message

**Table 4:** Lifetime metrics of the sensor node.

	LEACH [11]	LEACH-C [17]	CEEC [31]	HUCL [32]	SEED [33]	NEHCP [25]	HCEH-UC
First node dead	452	513	1000	1250	1510	1756	2535
Half node dead	534	555	1980	2510	3530	4100	4481
Last node dead	621	740	2675	3120	4200	4912	5145
Average	535.7	602.7	1885	2293.3	3080	3589.3	4053.7

**Table 5:** MLC under a different initial energy.

Initial-Energy	LEACH [11]	LEACH-C [17]	CEEC [31]	HUCL [32]	SEED [33]	NEHCP [25]	HCEH-UC
0.25 J	336	347	1500	1775	2012	2500	2573
0.5 J	621	740	2675	3120	4200	4912	5145
0.75 J	844	946	4088	4512	5312	6075	7713
1 J	1133	1336	4912	5587	7500	8300	10,280

## Data Availability

Data are contained within the article.

## References

[1] Wang B. (2011). Coverage problems in sensor networks: A survey. ACM Comput. Surv..

[2] Parikh S., Vokkarane V.M., Xing L., Kasilingam D. Node-replacement policies to maintain threshold-coverage in wireless sensor networks. Proceedings of the 2007 16th International Conference on Computer Communications and Networks.

[3] Ju Q., Zhang Y. (2018). Predictive power management for internet of battery-less things. IEEE Trans. Power Electron..

[4] Sajwan M., Gosain D., Sharma A.K. (2019). CAMP: Cluster aided multi-path routing protocol for wireless sensor networks. Wirel. Netw..

[5] Shah B., Abbas A., Ali G., Iqbal F., Khattak A.M., Alfandi O., Kim K.I. (2020). Guaranteed lifetime protocol for IoT based wireless sensor networks with multiple constraints. Ad. Hoc. Netw..

[6] Liao W., Wu M., Wu Y. (2017). Design of multi-energy-space-based energy-efficient algorithm in novel software-defined wireless sensor networks. Int. J. Distrib. Sens. Netw..

[7] Dietrich I., Dressler F. (2009). On the lifetime of wireless sensor networks. ACM Trans. Sens. Netw..

[8] Zhang D., Li G., Zheng K., Ming X., Pan Z. (2014). An energy-balanced routing method based on forward-aware factor for wireless sensor networks. IEEE Trans. Ind. Inform..

[9] Manchanda R., Sharma K. (2020). Energy efficient compression sensing-based clustering framework for IoT-based heterogeneous WSN. Telecommun. Syst..

[10] Zhang J., Lin Z., Tsai P., Xu L. (2020). Entropy-driven data aggregation method for energy-efficient wireless sensor networks. Inf. Fusion.

[11] Heinzelman R.H., Chandrakasan A., Balakrishnan H. Energy-efficient communication protocol for wireless microsensor networks. Proceedings of the 33rd Annual Hawaii International Conference on System Sciences.

[12] Chen M., Leung V.C.M., Mao S., Yuan Y. (2007). Directional geographical routing for real-time video communications in wireless sensor networks. Comput. Commun..

[13] Pantazis N.A., Nikolidakis S.A., Vergados D.D. (2013). Energy-efficient routing protocols in wireless sensor networks: A survey. IEEE Commun. Surv. Tutor..

[14] Al-Karaki J.N., Kamal A.E. (2004). Routing techniques in wireless sensor networks: A survey. IEEE Wirel. Commun..

[15] Lindsey S., Raghavendra C.S. PEGASIS: Power-efficient GAthering in sensor information systems. Proceedings of the IEEE Aerospace Conference.

[16] Gawade R.D., Nalbalwar S.L. (2016). A Centralized energy efficient distance based routing protocol for wireless sensor networks. J. Sens..

[17] Heinzelman W.B., Chandrakasan A.P., Balakrishnan H. (2002). An application-specific protocol architecture for wireless microsensor networks. IEEE Trans. Wirel. Commun..

[18] Lindsey S., Raghavendra C., Sivalingam K.M. (2002). Data gathering algorithms in sensor networks using energy metrics. IEEE Trans. Parallel Distrib. Syst..

[19] Sah D.K., Amgoth T. (2020). Renewable energy harvesting schemes in wireless sensor networks: A Survey. Inf. Fusion.

[20] Ibrahim H.H., Singh M.S.J., Al-Bawri S.S., Islam M.T. (2020). Synthesis, Characterization and Development of Energy Harvesting Techniques Incorporated with Antennas: A Review Study. Sensors.

[21] La Rosa R., Livreri P., Trigona C., Di Donato L., Sorbello G. (2020). Strategies and Techniques for Powering Wireless Sensor Nodes through Energy Harvesting and Wireless Power Transfer. Sensors.

[22] Pereira F., Correia R., Pinho P., Lopes S.I., Carvalho N.B. (2020). Challenges in Resource-Constrained IoT Devices: Energy and Communication as Critical Success Factors for Future IoT Deployment. Sensors.

[23] Rathore R.S., Sangwan S., Prakash S., Adhikari K., Kharel R., Cao Y. (2020). Hybrid WGWO: Whale grey wolf optimization-based novel energy-efficient clustering for EH-WSNs. EURASIP J. Wirel. Commun. Netw..

[24] Zeadally S., Shaikh F.K., Talpur A., Sheng Q.Z. (2020). Design architectures for energy harvesting in the Internet of Things. Renew. Sustain. Energy Rev..

[25] Sah D.K., Amgoth T. (2020). A novel efficient clustering protocol for energy harvesting in wireless sensor networks. Wirel. Netw..

[26] Khademi Nori M., Sharifian S. (2020). EDMARA2: A hierarchical routing protocol for EH-WSNs. Wirel. Netw..

[27] Nisha U.N., Basha A.M. (2020). Triangular fuzzy-based spectral clustering for energy-efficient routing in wireless sensor network. J. Supercomput..

[28] Thiagarajan M.R. (2020). Energy consumption and network connectivity based on Novel-LEACH-POS protocol networks. Comput. Commun..

[29] Mina N., Singh U., Salgotra R., Bansal M. (2020). An energy-efficient stable clustering approach using fuzzy-enhanced flower pollination algorithm for WSNs. Neural Comput. Appl..

[30] Behera T.M., Mohapatra S.K., Samal U.C., Khan M.S., Daneshmand M., Gandomi A.H. (2020). I-SEP: An Improved Routing Protocol for Heterogeneous WSN for IoT-Based Monitoring. IEEE Internet Things J..

[31] Aslam M., Shah T., Javaid N., Rahim A., Rahman Z., Khan Z.A. CEEC: Centralized energy efficient clustering a new routing protocol for WSNs. Proceedings of the 2012 9th Annual IEEE Communications Society Conference on Sensor, Mesh and Ad Hoc Communications and Networks (SECON).

[32] Malathi L., Gnanamurthy R.K., Chandrasekaran K. (2015). Energy efficient data collection through hybrid unequal clustering for wireless sensor networks. Comput. Electr. Eng..

[33] Ahmed G., Zou J., Fareed M.M.S., Zeeshan M. (2016). Sleep-awake energy efficient distributed clustering algorithm for wireless sensor networks. Comput. Electr. Eng..

[34] Dror E., Avin C., Lotker Z. (2013). Fast randomized algorithm for 2-hops clustering in vehicular ad-hoc networks. Ad. Hoc. Netw..

